# Rational Control of Molecular Properties Is Mandatory to Exploit the Potential of PROTACs as Oral Drugs

**DOI:** 10.1021/acsmedchemlett.1c00298

**Published:** 2021-06-08

**Authors:** Giuseppe Ermondi, Diego Garcia Jimenez, Matteo Rossi Sebastiano, Giulia Caron

**Affiliations:** Molecular Biotechnology and Health Sciences Dept., CASSMedChem, University of Torino, via Quarello 15, 10135 Torino, Italy

**Keywords:** bRo5, chameleonicity, intramolecular interaction, permeability, property-based drug design, PROTAC

## Abstract

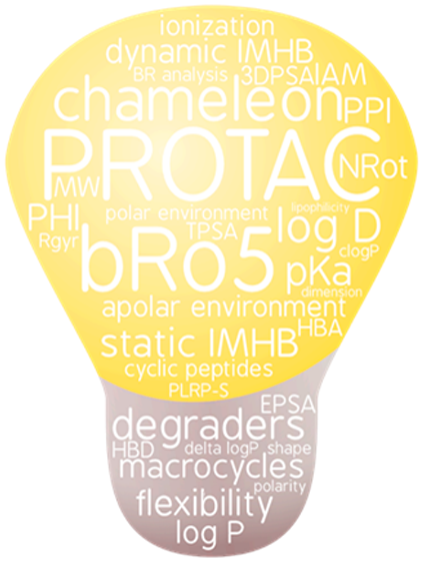

To obtain new oral drugs in the beyond rule of five space, PROTACs among others, molecular properties should be optimized in early drug discovery. Degraders call for design strategies which focus on intramolecular interaction and chameleonicity. In parallel, tailored revalidation of permeability assessment and prediction methods becomes fundamental in this innovative chemical space.

Heterobifunctional degraders^1^ (often named PROTACs) consist of a warhead that binds a protein of interest (POI), a linker, and a ligand that recruits an E3 ubiquitin ligase (Figure 1A).^1^ These molecules bring a POI close to the E3 ligase, triggering the target ubiquitination and subsequent degradation.^1^ From a chemical point of view, PROTACs may include cyclic peptides, macrocycles, and non-macrocyclic substructures. Moreover, degraders are expected to dominate the clinical trial population over the next years,^2^ since two oral PROTACs (ARV-110 and ARV-471, Figure 1B) recently reached Phase 2 clinical trials.^2^

**Figure 1:**
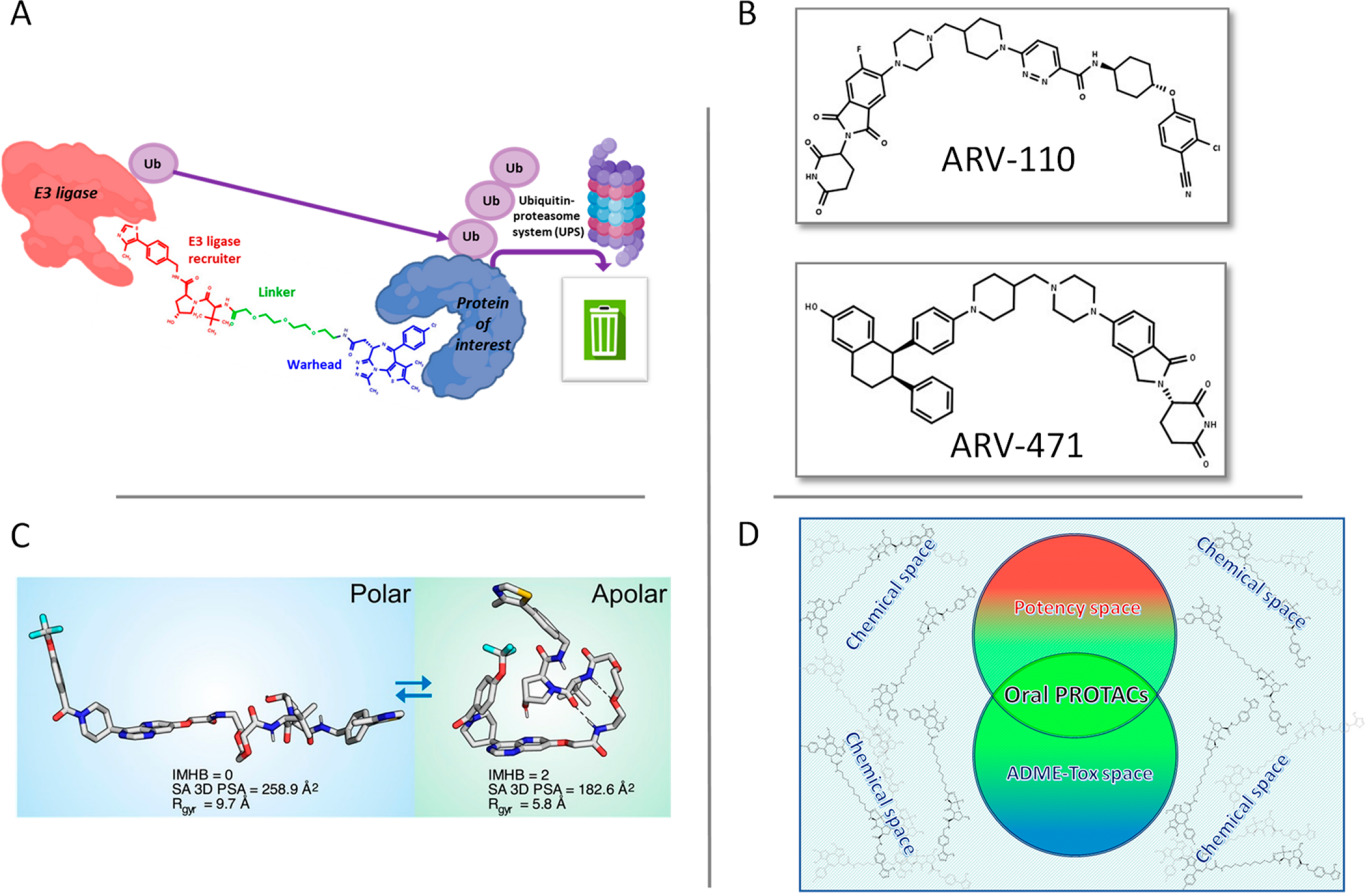
(A) PROTAC building blocks. (B) Chemical structures of PROTACs in Phase 2 clinical trials. (C) Environment dependent conformations of a potential VHL-based anticancer PROTAC (Adapted from ref (6). Copyright The Authors. CC BY 4.0.) (D) Chemical space in which active candidates become oral drugs.

Protein degraders and other new chemical modalities (e.g., RNA therapeutics, antibody–drug conjugates, and gene therapy) lie in the chemical space outside of Lipinski’s rule of five, termed beyond the rule of five (bRo5). PROTACs often exhibit higher specificity, potency, long duration of action, but also limited tissue penetration and issues with oral delivery. However, the number of molecules approved in this chemical space significantly increased in the last years.^3^

Protein degraders (and bRo5 compounds in general) are unlike what medicinal chemists like to work with; they exhibit large and flexible structures that can form intramolecular hydrogen bonds (IMHBs)^4^ and other conformer- and environment-dependent intramolecular interactions.^5^ These structural features allow PROTACs to adapt their properties to the environment. For instance, Kihlberg and co-workers verified that a potential VHL-based anticancer PROTAC^6^ is cell permeable and populates different conformations depending on the solution environments. Specifically, extended and polar conformers are present in water, whereas folded and less polar conformations are found in chloroform, a nonpolar environment often used to simulate the cell membrane interior (Figure 1C). Therefore, the study confirms that PROTACs could behave as molecular chameleons. Overall, this and other studies provide evidence that permeable and thus bioavailable PROTACs can be obtained if the impact of specific structural motifs on molecular properties (and thus on ADME profile) is properly controlled and implemented in design strategies.

Since PROTAC became a very hot topic in drug discovery, several online discussion corners have been organized in the last year. Unfortunately, after attending a few meetings on PROTACs, one can easily realize that property-based drug design is very poorly considered. Moreover, lessons from previous small molecules drug discovery campaigns have apparently been forgotten.^7,8^ In the past, many bench-active small molecules were designed regardless of their effective pharmacokinetic profile. This led to the pre- and clinical failure of most of them due to low bioavailability, with the consequent waste of resources. Unfortunately, PROTACs seem to follow the same trend. In fact, most, if not all pharma and biotech researchers are synthesizing hundreds of *in vitro* bioactive degraders and chemical probes (toolkits for PROTAC chemical synthesis are commercially available) which could hardly become oral drug candidates (Figure 1D) because of the lack of an adequate ADME profile. In meeting presentations, efforts are put in the disclosure of many structures, focusing on degradation activity and sometimes on the characterization of binary and ternary complexes with target proteins, regardless of physicochemical data. For instance, the widely known log *D* in the octanol/water system is not often shown with the structures. Notably, the lack of molecular properties is common in most PROTAC-related papers, not only in those reported in chemical biology journals but also in medicinal chemistry publications, supposedly caring for the pharmacokinetics (PK) of future drug candidates. Moreover, even highly evolved proprietary platforms (e.g., C4 Torpedo and Kymera Pegasus) which implement sophisticated strategies to predict ternary complex formation and PKPD models do not include any tools to obtain reliable physicochemical descriptors (which lipophilicity descriptors are included in the PKPD model?).

Which are the reasons for this trend? We hypothesized as follows: first, in commercial institutions, the quality of a project is judged (among others) by the number of produced compounds. Second, PROTAC scientists often have a molecular biology background and thus are not familiar with property-based drug discovery strategies. Finally, medicinal chemists do not know how to manage property-based drug design in the bRo5 chemical space.^9^ To better analyze this last aspect, we need to recall that property-based drug design (or molecular property design) is the approach that allows optimizing drug candidates by modulating molecular properties in early drug discovery. Molecular property design often combines sets of physicochemical descriptors in rules of thumb, with the rule of five (Ro5) being the most widely known, to guide the synthesis of better candidates. The rationale of this medicinal chemistry strategy is based on two assumptions: (a) drugs occupy a subset of the entire chemical space (Figure 1C) and (b) physicochemical properties are the major determinants of permeability, solubility, and *in vitro* ADME properties.

Given numerous violations being brought to light by retrospective studies focusing on the limits of Ro5 predictivity,^3^ a lot of discussion about the goodness of those rules of thumb has been reported in the recent medicinal chemistry literature (a summary is beyond the aim of the paper). In our opinion, an optimization of physicochemical properties through reasonable criteria drives the design of better oral drug candidates with an expected acceptable ADME profile and could be a good support to their synthesis. However, it is now evident that a direct transfer of strategies from Ro5 to bRo5 is not feasible. In fact, descriptors commonly used for Ro5 compounds cannot be applied to the bRo5 chemical space (Figure 2A) for at least three reasons: (a) they do not consider the 3D structure of molecules, (b) no descriptor is related to a nonpolar environment, an aspect needed to include chameleonicity, and (c) flexibility cannot be treated using simple descriptors such as the number of rotatable bonds (NRot), which also fails in the presence of cyclic substructures.

**Figure 2:**
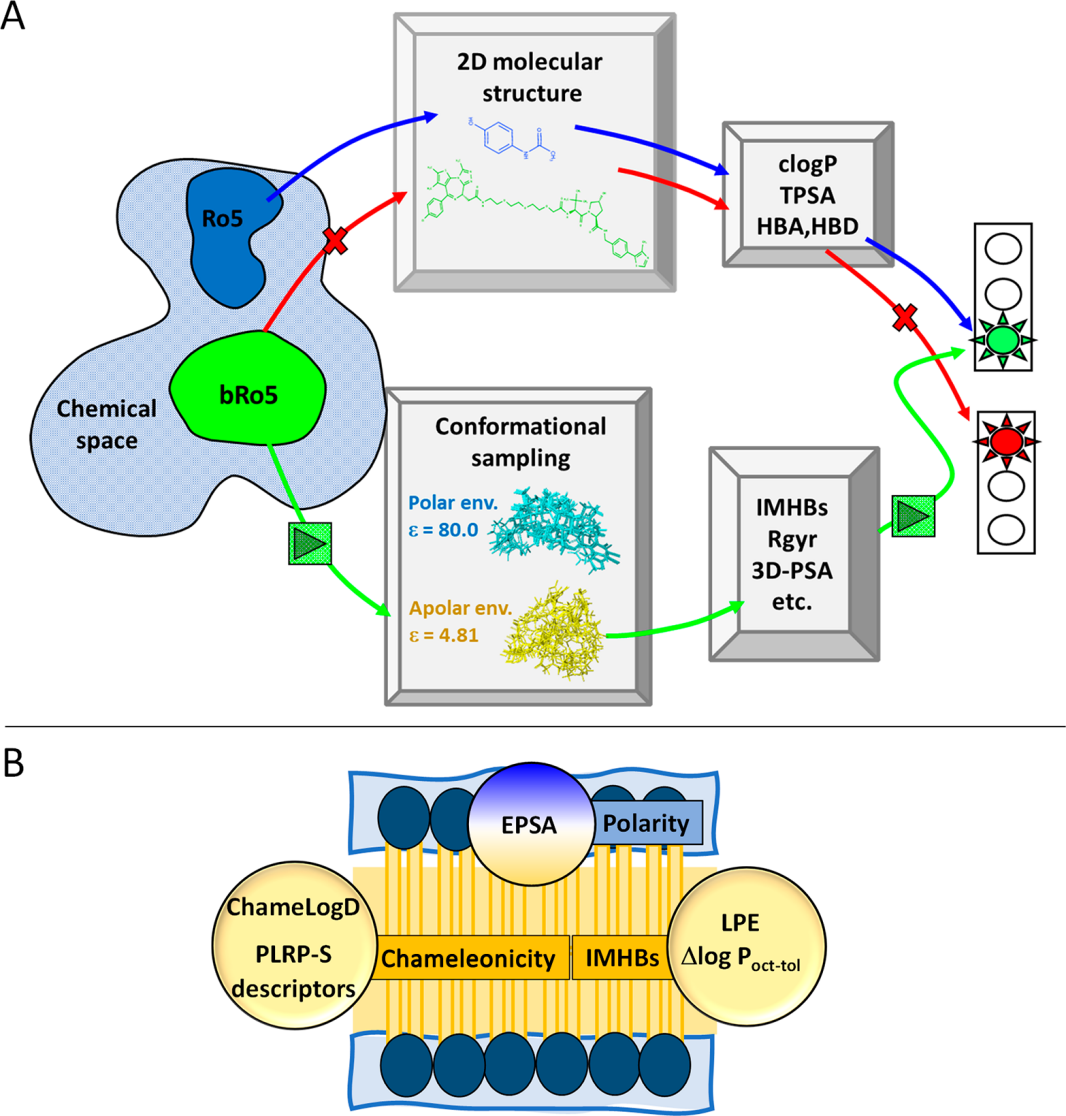
bRo5 descriptors of relevance in property-based drug design: (A) in silico and (B) experimental.

Overall, drug discovery strategies that manage to properly modulate molecular properties are not yet available for PROTACs. Therefore, there is the need for an update of descriptors tailored to large and flexible compounds that would allow, based on descriptor thresholds, one to identify the most promising PROTACS.

Permeability measurement methods for early screening need to be carefully validated in the bRo5 space, including the assessment of the relevance of efflux transporters and the limits due to molecular weight.^10^ Available data (often produced by pharma companies and in large part undisclosed) seem to suggest that permeability is best defined by cell-based assays, although nonspecific binding, which also affects mass balance recovery, should be addressed. The parallel artificial membrane permeability assay (PAMPA) seems to be less suitable for PROTACs, but PAMPA methods that do not use dodecane could perform better. Permeability assays based on tagging procedures are not suggested at least in early drug discovery since tags modify the PROTAC structure and thus their properties. In addition, a direct link between bioavailability data and physicochemical descriptors could be established and be of great help in drug design. This of course is feasible once enough degraders spanning a good bioavailability range will be available.

As discussed above, to obtain promising bRo5 drug candidates, *ad hoc* physicochemical descriptors should be determined in early drug discovery. This would enable a reliable estimation of permeability and *in vitro* ADME properties. Researchers are working to figure out which descriptors (and assays) are appropriate to be applied within the bRo5 and thus the PROTAC compound space. Recently, we proposed a pool of physicochemical properties^11^ that are divided into two groups: general properties valid for any drug candidate and properties specific for bRo5 compounds. All drugs and candidates can be described with general molecular properties like size and shape, ionization, lipophilicity, and polarity. Notably, most molecular properties can be quantified by different descriptors. For instance, lipophilicity can be quantified by log *P*/log *D* determined in various biphasic systems, with octanol/water being the most common but considering that toluene/water and its surrogates are becoming more and more relevant. Additional specific properties need to be quantified by *ad hoc* descriptors, since large and flexible structures can be represented by different conformers with different molecular properties (negligible feature in the Ro5 space). For instance, flexibility and hydrogen bond properties call for new descriptors able to quantify PROTAC propensity to form IMHBs but also other intramolecular interactions.

Since property-based drug discovery can be applied at different drug discovery stages, both computed and experimental descriptors are required. *In silico* strategies in the bRo5 space (Figure 2A) are significantly different from Ro5 (essentially based on 2D calculators) and involve the generation of averaged 3D structures by conformational sampling in polar and nonpolar environments and calculation for any conformer of molecular descriptors like radius of gyration (Rgyr), polar surface area (3D-PSA), number of IMHBs, and chameleonicity indexes. About the conformational sampling, we need to recall that more than one method should be utilized, since specific force-field-related issues in energy calculation cannot be neglected (their exhaustive discussion is beyond the aim of this viewpoint). Consequently, property distribution inspection allows one to identify potential chameleons and/or conformers with a unique property profile. The second main goal of conformational analysis is the identification of biorelevant conformations to be used in the generation of statistical models for permeability and *in vitro* ADME properties. A reasonable proposal consists of the identification for any PROTAC of a pool of conformers of potential impact. For instance, the most lipophilic and the less polar conformers in nonpolar media could be used for permeability modeling, whereas the most polar conformer in water could account for solubility. These criteria are suggested to be integrated with considerations about the energetic price to pay when passing from one environment to the other (congruent conformations).^12^

Experimental descriptors of relevance in the bRo5 space may be of different nature, with chromatographic methods based on gradient conditions not recommended since they produce an environment variation during the experiment. Overall, there is a consensus about a few promising descriptors: (a) EPSA to quantify molecular polarity,^13^ (b) Δlog *P*_oct-tol_ (the difference between log *P* in octanol/water and log *P* in toluene/water)^14^ and its analogue LPE (lipophilicity permeability efficiency, defined as the difference between calculated ALOGP and log *P* measured in the decadiene/water system)^15^ to monitor the presence of dynamic IMHBs, and (c) ChameLogD (the difference between ElogD and BRlogD)^16^ and descriptors obtained from nonpolar chromatographic systems based on the PLRP-S column, as chameleonicity quantifiers.^17^ Notably, all these descriptors either monitor molecular properties in nonpolar media or compare molecular behavior in environments with different polarity and are expected to be major determinants of permeability (Figure 2B). bRo5 experimental physicochemical data published up to now are proofs of concept to highlight the contribution of specific structural features involved in intramolecular interactions to permeability.^16,9^ However, their limited number does not allow the setup of general rules of practical application neither in PROTAC nor in other research programs. Nevertheless, from the analysis of available bRo5 specific physicochemical descriptors, some comments are feasible. First, validation sets are still missing. PROTAC structures are in fact extremely different in terms of building blocks, and generalization are not allowed. Second, even though the formation of IMHB does not always induce chameleonicity, a relationship between the number of IMHBs (static and dynamic) and permeability exists and chameleonicity itself impacts permeability and bioavailability. Finally, the disclosure of the chemical structure of Arvinas compounds in clinical trials strongly supports the major role played by the linker in intramolecular interaction modulation and calls for more investigation inside series sharing the same warhead and E3 ligand but varying in linker structures.

Lessons learned from previous drug discovery campaigns should be understood and taken in mind to improve PROTAC technology and obtain new oral drugs instead of just chemical probes. In particular, an efficient property-based drug design is needed to obtain drug candidates with an expected acceptable ADME profile. However, efforts should be made to define descriptors and strategies tailored to large and flexible structures, significantly different from the traditional Ro5 chemical space.

The application domain of existing approaches to measure permeability like PAMPA and Caco-2 (but also lipophilicity descriptors) should be verified and updated, whereas new computational and experimental methods should be designed and implemented in order to control the impact of specific bRo5 structural features like IMHB and chameleonicity on ADME properties. In this respect, conformational sampling in different environments and conformer property calculations are expected to provide tools to predict intramolecular interactions and identify biorelevant conformers to be used in modeling studies for permeability prediction. From an experimental point of view, interesting descriptors like EPSA, ChamelogD, and others should be extensively measured (Figure 2B).

Due to the high number of synthesized (and uncharacterized) compounds that have not yet been fully disclosed by industrial partners and the high know-how capital in terms of structure–property prediction in academia, collaborative sharing of knowledge and materials between these two worlds is the key to success. Hopefully, collaborative efforts between academia and industry will accelerate the process of data collection and allow the generation of validated filtering procedures enabling an efficient PROTAC drug candidate prioritization.
